# Synthesis and Characterization of Magnetic Composite Theragnostics by Nano Spray Drying

**DOI:** 10.3390/ma15051755

**Published:** 2022-02-25

**Authors:** Caio José Perecin, Xavier Pierre Marie Gratens, Valmir Antônio Chitta, Patrícia Leo, Adriano Marim de Oliveira, Sérgio Akinobu Yoshioka, Natália Neto Pereira Cerize

**Affiliations:** 1 São Carlos Institute of Chemistry, University of São Paulo, São Carlos 13566-590, SP, Brazil; sergioy@iqsc.usp.br; 2 Bionanomanufacturing Center, Technological Research Institute, São Paulo 05508-070, SP, Brazil; patrileo@ipt.br (P.L.); amarim@ipt.br (A.M.d.O.); ncerize@ipt.br (N.N.P.C.); 3 Institute of Physics, University of São Paulo, São Paulo 05508-090, SP, Brazil; xgratens@gmail.com (X.P.M.G.); vchitta@if.usp.br (V.A.C.)

**Keywords:** magnetic nanoparticles, theragnostic, magnetic hyperthermia, composites, nano spray drying, superparamagnetism

## Abstract

Composites of magnetite nanoparticles encapsulated with polymers attract interest for many applications, especially as theragnostic agents for magnetic hyperthermia, drug delivery, and magnetic resonance imaging. In this work, magnetite nanoparticles were synthesized by coprecipitation and encapsulated with different polymers (Eudragit S100, Pluronic F68, Maltodextrin, and surfactants) by nano spray drying technique, which can produce powders of nanoparticles from solutions or suspensions. Transmission and scanning electron microscopy images showed that the bare magnetite nanoparticles have 10.5 nm, and after encapsulation, the particles have approximately 1 μm, with size and shape depending on the material’s composition. The values of magnetic saturation by SQUID magnetometry and mass residues by thermogravimetric analysis were used to characterize the magnetic content in the materials, related to their magnetite/polymer ratios. Zero-field-cooling and field-cooling (ZFC/FC) measurements showed how blocking temperatures of the powders of the composites are lower than that of bare magnetite, possibly due to lower magnetic coupling, being an interesting system to study magnetic interactions of nanoparticles. Furthermore, studies of cytotoxic effect, hydrodynamic size, and heating capacity for hyperthermia (according to the application of an alternate magnetic field) show that these composites could be applied as a theragnostic material for a non-invasive administration such as nasal.

## 1. Introduction

Theragnostic materials emerge as powerful tools for medicine, taking advantage of the properties of nanostructures to provide therapeutic and diagnostic functions to act in specific targets of the organism [1,2]. Superparamagnetic iron oxide nanoparticles (SPIONs) of magnetite or maghemite are good examples of building blocks to produce materials with theragnostic capacities. They present adequate biocompatibility and magnetic properties to be used for numerous in vivo applications, such as hyperthermia to treat cancer [3], drug delivery by magnetic targeting [4], and contrast agents for the diagnosis of diseases by magnetic resonance imaging (MRI) [5], but also for other purposes, such as detoxification of liquids, cell separation [6], and sensing systems [7].

In particular, magnetic hyperthermia is a type of cancer treatment based on the heat generated by SPIONs under an alternate magnetic field, to increase the local temperature to 42–46 °C, which induces the death of tumor cells [3,8]. The therapy has been improved over the last decades especially by enhancing the capacity of the nanoparticles to produce heat [9], however, it is a challenge to target the particles at the tumor site through a non-invasive route of administration and treat it effectively [10,11].

Nasal administration of powders of therapeutics is appealing as it is a painless route, allows self-administration, gives rapid onset of action, and avoids the first-pass metabolism, limiting drug degradation. The alveolar region of the lung offers a huge surface area (approximately 100 m^2^) for systemic absorption of the drug with only a thin epithelial barrier and is free of mucus and cilia [12,13,14]. However, the drawbacks are that inhalation of chemotherapeutics can cause pulmonary toxicity due to high concentration locally and are rapidly eliminated, which requires multiple daily administrations. Drug delivery systems of nano- and micro-particles can circumvent these issues since they can promote sustained release and reportedly avoid mucociliary clearance and lung phagocytic mechanisms [15]. For example, magnetic nanomaterials coated with polymers have shown promising results as lung cancer theragnostics [16,17].

In fact, all biomedical applications require peculiar surface coatings of the SPIONs, which also must be nontoxic and biocompatible, to stabilize them against aggregation and oxidation [18], as well as to functionalize them for specific tasks [19]. Encapsulation with appropriate polymers is a good path to obtain composite materials that combine the properties of inorganic and organic units [20].

One can explore the vast library of pre-formed polymers, depending on the specific application. Eudragit^®^ S 100 is an anionic copolymer of methacrylic acid and methyl methacrylate (1:2), developed for the controlled release of drugs. Since it is sensitive to pH, being soluble/degraded only at a basic pH, it is suitable for the encapsulation of bioactive molecules for drug delivery to the intestine after oral administration [21]. Maltodextrin is a polysaccharide formed by D-glucose units. Carbohydrates, such as maltodextrin, play important roles in several biological processes, such as cell signaling, cell adhesion, cell migration, and in the development and metastasis of tumors, are able to act as markers for nanoparticles to reach molecular targets. The presence of lactose and glucose on the surface of particles may increase their internalization in tumor cells [18]. These molecules can also promote the passage through biological barriers, such as the plasmatic membrane and the blood–brain barrier [22]. Tween 80, a non-ionic hydrophilic tensoactive, is widely employed for encapsulation to act as an emulsifier, helping to stabilize the system. Poloxamer 188, a three-block copolymer of poly(ethylene oxide)-poly(propylene oxide)-poly(ethylene oxide) (PEO-PPO-PEO), is reported to cause drastic sensitization of MDR (Multiple Drug Resistance) tumors to anticancer agents [23]. Both Tween and Poloxamer are also able to enhance the ability to enhance drug transport across biological barriers [24,25].

Spray drying is a well-established strategy to produce powders from solutions or suspensions [26]. The nano spray drying version takes the advantages of regular spray dryers, such as simplicity, scalability, and product stability, yet distinctly obtains smaller particles, in the size range of few microns and sub-micron [27]. The technique is versatile and can be used to obtain materials with varied compositions, such as for the encapsulation or coating of solid nanoparticles with polymers, also called nanoenglobing. Regarding biomedical applications, it is an interesting method to produce powders of nanoparticles to be administrated via nasal or oral routes [28,29].

In this work, magnetite nanoparticles were synthesized by a co-precipitation method and encapsulated by Nano Spray Drying with different polymers. The technique enables the production of submicron particles by exploring a piezoelectric driven vibrating mesh atomizer that creates tiny droplets smaller than in classical spray dryers and their collection through a high-efficiency electrostatic dry powder collector. With this strategy, the aim was to propose a versatile protocol to produce nanostructured magnetic composites for biomedical applications, focusing on magnetic hyperthermia.

## 2. Materials and Methods

### 2.1. Materials

Iron (III) chloride hexahydrate (FeCl_3_·6H_2_O), Iron (II) chloride tetrahydrate (FeCl_2_·4H_2_O) were purchased from Sigma-Aldrich (São Paulo, SP, Brazil), Ammonium hydroxide solution (NH_3_) 25% from Merck (São Paulo, SP, Brazil), Eudragit^®^ S100 (M_W_ = 125,000) from Evonik Industries (Darmstadt, Germany), Maltodextrin from Givaudan (São Paulo, SP, Brazil) and Tween 80 (Polysorbate 80, M_W_ = 1310) from Mapric Produtos Farmacosméticos (São Paulo, SP, Brazil).

### 2.2. Synthesis of Magnetite Nanoparticles

Four syntheses of magnetite were performed by co-precipitation method, first developed by Massart et al. [30], with some modifications. First, 1.99 g of FeCl_2_·4H_2_O and 4.05 g of FeCl_3_·6H_2_O were solubilized in 200 mL of water, either with 1 g of Eudragit S100 (ME), Pluronic F68 (MP), Tween 80 (MT), or without polymer (Mag), depending on the synthesis protocol. The solution was added to a round bottom flask at room temperature (21 °C), under ultrasonication, mechanical stirring, and N_2_ purging. Then, 50 mL of NH_4_OH 25% was added dropwise for 5 min and the system was kept under stirring for more 40 min. The product was washed three times by centrifugation and resuspension in deionized water.

### 2.3. Encapsulation by Nano Spray Drying

The encapsulation was performed with the Nano Spray Drying equipment (BUCHI) [31]. The feeding fluids for the equipment were suspensions of magnetite nanoparticles as they were synthesized or mixed with more polymers, in the compositions described in Table 1:

The fluids that had the addition of more polymers were submitted to mechanical stirring of approximately 800 rpm for 15 min to homogenize the suspension. All the fluids were kept under mild stirring (approximately 150 rpm) in a beaker as they were fed to the equipment.

To start the drying process, a peristaltic pump takes the fluid from the beaker to the spray head through a silicon feeding tube. In the spray head, the fluid passes through a vibrating micropore membrane (60 Hz) to produce fine droplets into the drying cylinder (150 cm long). A hot air flow enters the top of the cylinder to dry the droplets along it and an electrical particle collector in the bottom retrieves the dried particles by an electrostatic field. Finally, after the equipment is turned off, the particles are collected manually from the particle collector.

The equipment drying parameters were set as: membrane of 5.5 µm pore size, inlet temperature of 120 °C, outlet temperature of 54 °C, and a gas flow of 130 L/min.

### 2.4. Characterization

Particle size and morphology were verified by Transmission Electron Microscopy (TEM), using a JEOL JEM2100 microscope (Peabody, MA, USA) operating at 200 kV, and by Scanning Electron Microscopy (SEM), using a FEI Quanta 3D high resolution microscope SEM-FEG (Hillsboro, OR, USA). For TEM, particles were dispersed in isopropanol and dropped onto copper grids coated with Formvar^®^ and carbon films; for SEM, sample powders were deposited in an adhesive copper strip and were metalized with a thin gold layer.

X-ray diffractometry (XRD) was performed with a Shimadzu XRD-6000 (Kyoto, Japan). The dried samples were prepared by macerating and compacting them in a sample holder. The X-ray patterns were collected between 20° and 80° in 2θ using the Cu Kα radiation.

Fourier transform infrared spectroscopy (FTIR) was performed using pellets of a mixture of the dried nanoparticles powder with KBr, with wavelengths between 400 and 4000 cm^−1^, in a Shimadzu IRAffinity-1 equipment (Kyoto, Japan). The results were normalized.

Thermogravimetric Analysis (TGA) curves were obtained with a Mettler Toledo TGA/DSC 1 analyzer (Columbus, OH, USA), from 25 °C to 1000 °C, with a N2 flow of 80 mL/min.

Hydrodynamic size distributions were determined employing dynamic light scattering (DLS) technique using a Zetasizer Nano ZS (Malvern Instruments, Malvern, UK). Nanoparticles were dispersed in distilled water.

The magnetization measurements were obtained with a Cryogenic S600 SQUID Magnetometer (London, UK). For the Zero-field-cooling experiment, the sample was cooled down to 2 K without an external magnetic field, then a magnetic field of 150 Oe was applied, and the magnetization was registered during the heating of the sample up to 300 K. For the Field-cooling experiment, the sample was cooled down to 2 K in a 150 Oe magnetic field, and then its magnetization was measured during the heating, under the same magnetic field. The blocking temperature (T_B_) was determined as the maximum value of the ZFC curve. For magnetization (emu per gram of sample) vs. applied magnetic field H measurements, particles were kept at 1.7 K and 300 K, while H varied from −65 to +65 kOe.

The biological tests of cell viability in the presence of the particles were performed through neutral red uptake assay according to ISO 10993-5, using NCTC-929 (mouse fibroblasts), HeLa (human cervical adenocarcinoma), and HepG2 (human hepatocellular carcinoma) cell lines. The cells were incubated in 96-well microtiter plates (100 μL; 1 × 10^5^ cells/mL) and left to adhere for 24 h, after which the medium was removed. The particles were dispersed in phosphate-buffered saline (PBS) (0.137 mol/L) and diluted in fetal bovine serum (FBS) (5% m/m), to obtain suspensions of particles in the concentration range of 0.02 mg/mL to 7.6 mg/mL. The suspensions were added to the wells in six replicates and the negative control (FBS 5% without particles) of each cell line was performed in twelve replicates. After exposure of 24 h, the particles were removed and FBS 5% medium with neutral red dye (1 mg/mL) was added. After incubation of 3 h, the medium was exchanged by an ethanol/acetic acid/water (50%/1%/49%) solution to extract the dye and the absorbances at a wavelength of 540 nm were measured using a Titertek Multiskan plate spectrophotometer (McLean, VA, USA). The average absorbance of the negative control wells was considered as 100% viability and the effects of the particles on the cells were calculated relatively to it using the average results of their wells.

## 3. Results and Discussion

Composite nanoparticles of magnetite and different polymers were obtained through a versatile straightforward strategy by a chemical synthesis of magnetite and a physical method of encapsulation by nano spray drying. First, the synthesis of the magnetic nuclei was performed by coprecipitation, which is a widely used procedure to obtain hydrophilic magnetite nanoparticles. Pluronic F68, Tween 80, and Eudragit were added to the reaction medium, to obtain MP, MT, and ME. Besides them, a sample of bare (naked, without coating) magnetite nanoparticles, named Mag, was synthesized for comparison purposes. All these formulations generated stable black magnetic suspensions. Following this, the suspensions were prepared as they were or with the addition of more polymer (Table 1), and spray dried to form brownish powders MP-SD, ME-SD, M3E-SD, and MTM-SD. The use of a membrane of 5.5 µm pore that is vibrated at an ultrasonic frequency of 60 Hz allows the obtaining of sub-micron particles, through an automatized process.

**Figure 1:**
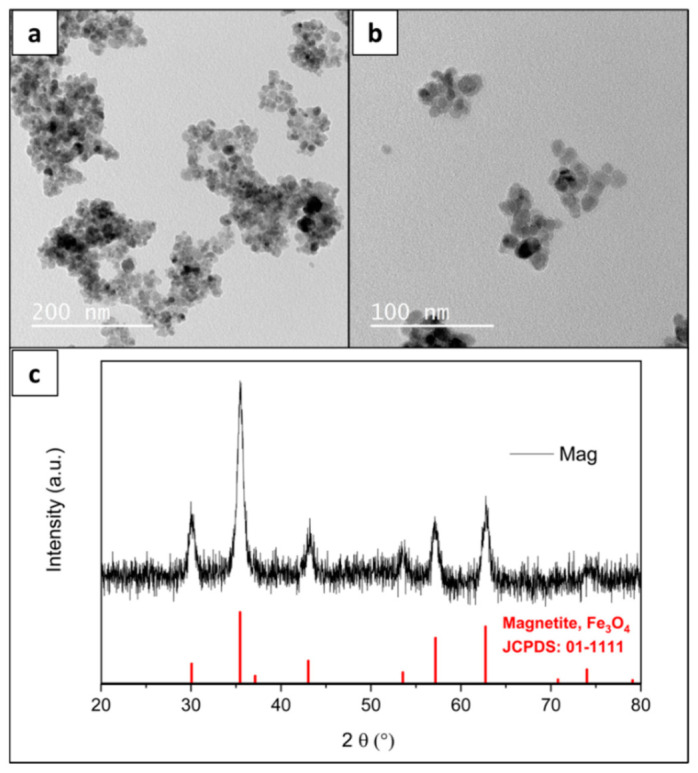
Transmission electron microscopy (TEM) images (**a**,**b**) and X ray diffractometry (XRD) (**c**) of the sample of bare magnetite Mag, with standard magnetite JCPDS 01-1111.

The diffractogram of bare nanoparticle Mag is presented in Figure 1c, showing the characteristic planes of the inverse spinel structure of magnetite [32], confirming the expected composition. TEM was performed to analyze the morphology of Mag and the images in Figure 1a,b show that the sample is composed of nanoparticles with approximately spherical shape in general, with a medium diameter of 10.5 nm. DLS analyses show that when the particles are dispersed in water, they are organized in aggregates with a Z-average (determined by the intensity of scattered light) of 206.6 nm and a number mean of 58.6 nm. The shape and size are typical of the coprecipitation route, as well as the slight aggregation of the suspension [20].

When the polymers are mixed with Mag suspensions, they adsorb upon these magnetite aggregates verified by DLS measurements. These mixture fluids were fed to the nano spray dryer, which can form particles of different characteristics depending on the aspects of the fluid, such as total concentration, the proportion of magnetite to polymer, and the character of the polymer. The drying conditions, such as temperature and membrane pore sizes, also have an important impact, however in these cases they were maintained at 120 °C (inlet gas temperature) and 5.5 µm, respectively.

**Figure 2:**
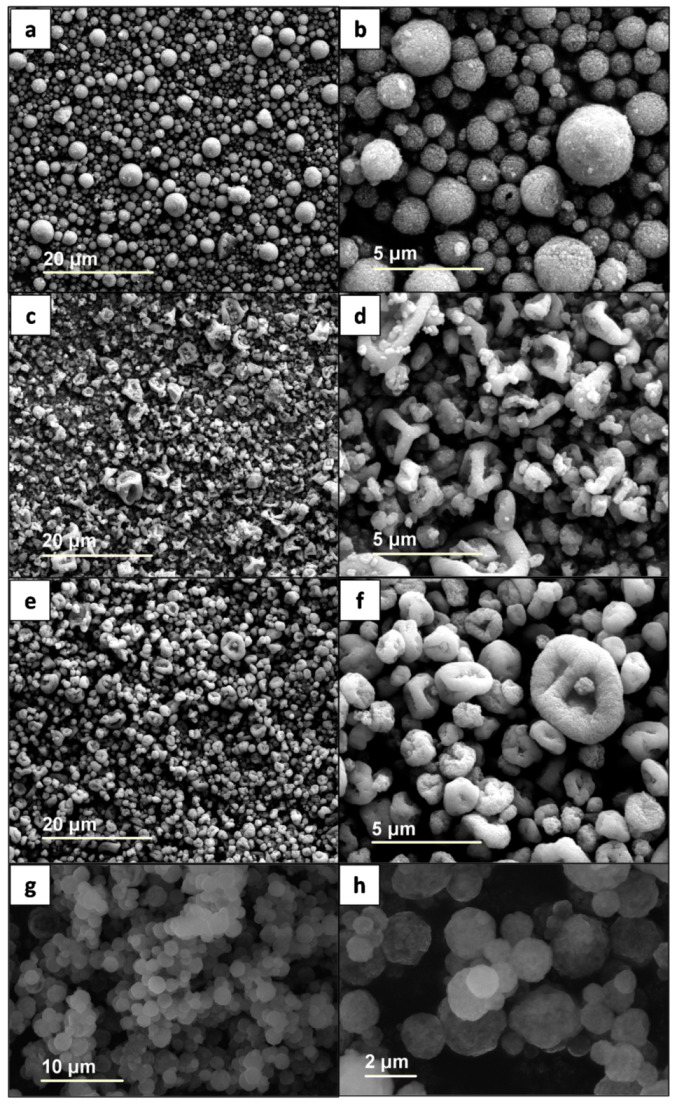
Scanning electron microscopy (SEM) images of MP-SD (**a**,**b**), ME-SD (**c**,**d**), M3E-SD (**e**,**f**), and MMT-SD (**g**,**h**) samples.

The images of SEM in Figure 2 depict the morphologies of the spray-dried particles of magnetite and polymers. The particles of MP-SD (Figure 2a,b) are approximately spherical, with a medium diameter of 1016 nm, similarly to M-3MT (Figure 2g,h), with 1155 nm. A closer look shows that the surface is not smooth and indicates that the magnetite is likely to be distributed across the whole nanostructure within the polymeric matrix, resembling a “3D nanocookie”. The diameters of ME-SD and M3E-SD (Figure 2c–f, respectively) vary between 500 and 3 μm, with averages of 899 nm and 1066 nm, respectively. Both samples with Eudragit have particles of irregular morphologies, especially the former, with hollow interiors.

The morphology of particles produced by spray drying of a nanoparticle suspension is affected by how the droplets are dried in the drying chamber; these toroid-like shapes seen in ME-SD and M3E-SD are associated with deformations of the droplets, differing from the regular spherical shape of MP-SD and MMT-SD [33]. As mentioned, the droplets’ formation is affected by the fluid composition (viscosity, size of the nanoparticles in suspension, presence of surfactants, and overall concentration) and the drying parameters (temperature and gas flow rate), however the latter were maintained fixed for all processes. The proportion of magnetite was similar between MP-SD and ME-SD; therefore, the shapes must be associated with the polymers’ characteristics. In fact, toroid-like shapes are typically seen in dried nanoparticles composed of Eudragit polymers, which can even form long filaments under specific drying conditions [34].

The particles were also dispersed in water and formed stable suspensions after approximately 10 min in an ultrasound bath. The hydrodynamic diameters are shown in Table 2 and are similar to the ones determined by SEM, except for MMT-SD, which has a dry diameter of 1155 nm and a hydrodynamic diameter of 288 nm. In this case, there is a high polymeric content of maltodextrin, which is hydrophilic and is readily solubilized in water, and the particles were probably dismantled to smaller agglomerates of magnetite and polymer. This formulation could be interesting for drug delivery, aiming for a fast drug release in the lungs after nasal administration, for example.

**Figure 3:**
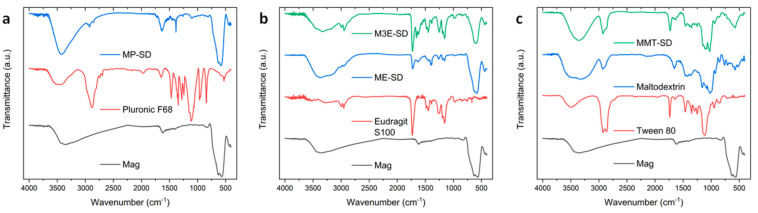
Fourier Transform Infrared Spectroscopy (FTIR) spectra of MP-SD (**a**), ME-SD (**b**), M3E-SD (**b**), and MMT-SD (**c**), compared to their constituent polymers and magnetite Mag.

FTIR results in Figure 3 confirm the chemical composition of the composite samples according to the reagents used. The spectrum of Mag shows the characteristic band of magnetite is present at 580 due to Fe-O stretch, with another band at 620 cm^−1^ that is associated with surface oxidation to maghemite [32]. Both bands are also present in the spectra of all the encapsulated samples. In MP-SD, these bands of iron oxide are very intense, with only discrete bands of Pluronic. Eudragit’s bands in M3E-SD are more intense than in ME-SD, as the former has more polymer content in its structure than the latter; on the other hand, iron oxide bands are more intense in ME-SD. Eudragit has its most intense band at 1730 cm^−1^, due to the C=O bonds of its ester and carboxylic acid functions, which are present in ME-SD and M3E-SD, as well as two bands at 2950 and 2997 cm^−1^ related to C-H bonds. In MMT-SD spectrum, the encapsulating matrix bands are more intense than those of magnetite, since the magnetite suspension (MT) was only 20% of the composition. The ketone band at 1730 cm^−1^ is present due to Tween 80; the large band between 3000 and 3700 cm^−1^ is related to O-H bonds in maltodextrin; the bands between 1000 and 1200 cm^−1^, present in maltodextrin and Tween 80, are attributed to C-O bonds. Bands at 2860 and 2960 cm^−1^ are related symmetric and asymmetric stretches of CH_2_, respectively.

**Figure 4:**
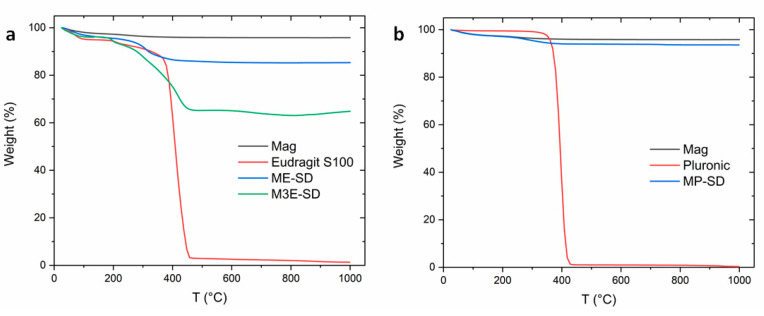
Thermogravimetric analyses (TGA) results of weight (%) with respect to temperature (°C) of samples ME-SD, M3E-SD (**a**), and MP-SD (**b**) in comparison to pure magnetite (Mag) and the polymers used Eudragit S100 and Pluronic.

According to the thermogravimetric analysis (TGA) reported in Figure 4, the bare magnetite sample has a residue of 95.8% until 1000 °C under nitrogen flow. In these conditions, it does not gain mass and the 4.2% mass loss is related to adsorbed water and impurities. Eudragit polymer loses almost all its mass remaining with a residue of 1.3% (Figure 4a) and Pluronic’s residue is negligible at 0.3% (Figure 4b). If the polymer residue is this low, the masses of polymer and magnetite in the encapsulated samples can be roughly estimated from their TGA curves, attributing their final residues mainly to iron oxide. The residues determined by TGA, associated with the magnetite contents in the composites, are shown in Table 3. A more precise determination can be performed by inductively coupled plasma (ICP), which was not performed in this study.

The TGA results of ME-SD (green curve) and M3E-SD (blue) samples show that they first lose 3.1% and 3.8% of water, respectively, until about 100 °C. After that, there is the degradation of the ester of the polymer’s side chain, followed by its main chain loss, with a peak of mass loss around 398 °C. M3E-SD had a final residue of 64.8% and 31% of polymer mass degraded, while ME-SD had a final residue of 85.3% and around 11.5% of polymer mass degraded. MP-SD had a higher residue, 93.6%. Even though both MP-SD and ME-SD did not have the addition of more polymer after magnetite synthesis by coprecipitation, differences in polymer content may arise from the amount of polymer that was retained in the surface of magnetite nanoparticles (or inside aggregates) during the washing process by centrifugation.

**Figure 5:**
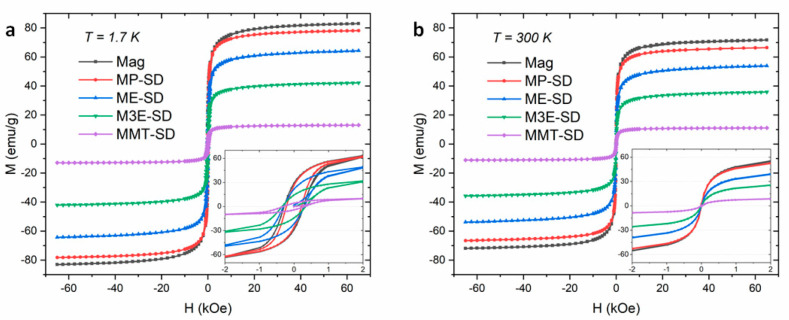
Hysteresis loops by M vs. H measurements at 1.7 K (**a**) and 300 K (**b**), with insets around H = 0 kOe.

Magnetic measurements of hysteresis loops performed at 300 K and 1.7 K are presented in Figure 5. The magnetic behaviors of the samples at 300 K are typical of superparamagnetic particles, with negligible coercivity, without hysteresis, and high response to the external field. In this condition, the thermal energy is able to make the magnetic moments constantly flip, resulting in a null net magnetization. At 1.7 K there is hysteresis, with coercivities around 300 Oe, in a ferrimagnetic state. This magnetization residual at H = 0 occurs due to the lack of thermal energy, allowing the magnetic moments to remain partially aligned. As the module of H increases, the module of M grows until it stabilizes at the saturation magnetization (M_S_), described in Table 3. The M_S_ values are different depending on the temperature also as a result of the occurrence of magnetic moment flips even at high H field. At 300 K, the moment orientation is less fixed, resulting in M_S_ values 13% to 16% smaller than at 1.7 K.

Among the samples, the magnetic saturation depends mainly on the proportion of magnetite in each of them. Mag had the highest values, 72.0 emu/g at 300 K and 83.1 emu/g at 1.7 K. The samples with polymers had lower values due to lower magnetite content, however, with regard to their suitability for application as a theragnostic, the saturations of MP-SD (68.7 emu/g) and ME-SD (53.9 emu/g) at 300 K are high considering an iron oxide composite material, indicating a good potential of performance. Furthermore, the saturation ratios between the composite samples and bare magnetite (M_S-Composite_/M_S-Mag_) could also be used to make a rough estimate of the magnetite content in them, which are presented in Table 3. For example, the M_S-Composite_/M_S-Mag_ of MP-SD is 95.5%, which differs from its TGA result by less than 2%.

**Figure 6:**
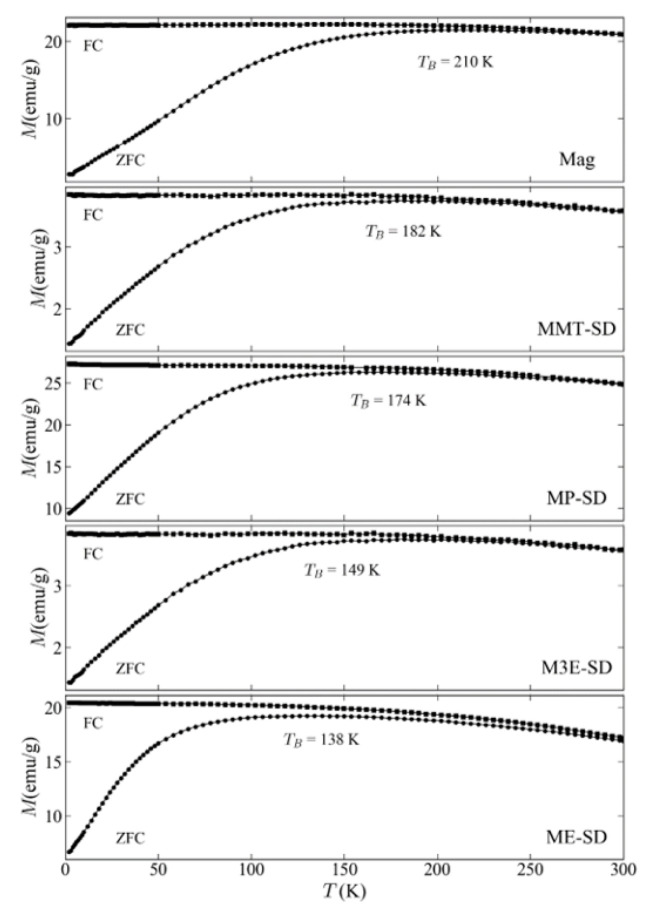
Zero-field-cooling and Field-cooling (ZFC/FC) measurements (magnetization vs. temperature) of the samples with the indication of the respective blocking temperature (T_B_).

Magnetic measurements versus temperature, ZFC/FC curves in Figure 6 allow a further investigation of the magnetic behavior of the samples, proving the superparamagnetic behavior at room temperature conditions. The temperature, from which the sample enters superparamagnetic regime, is the so-called blocking temperature (T_B_), that is determined as the highest value of ZFC curves. These are lower for the composites than for the naked magnetite. The T_B_ is directly proportional to the size of the magnetite nuclei [35], indicating that the use of polymers and surfactants in the synthesis may have lowered their sizes. This is probably due to the fact that the presence of these molecules on their surfaces inhibits their growth process, as reported previously [36]. Furthermore, there is certainly a change in the magnetic coupling depending on the average distance between the magnetite nuclei, which depends on the amount of polymer and the size of the whole particle. This might explain why ME-SD and M3E-SD have different T_B_ (138 K and 149 K, respectively) even though they are made of the same magnetic material, with different amounts of polymer. A higher amount of polymer would ensure more space between the nuclei of a particle, which would diminish magnetic coupling; on the other hand, M3E-SD had a larger particle size, enclosing more nuclei in a tighter space, enhancing coupling. The latter effect was more important in this case, since M3E-SD had a higher T_B_.

**Figure 7:**
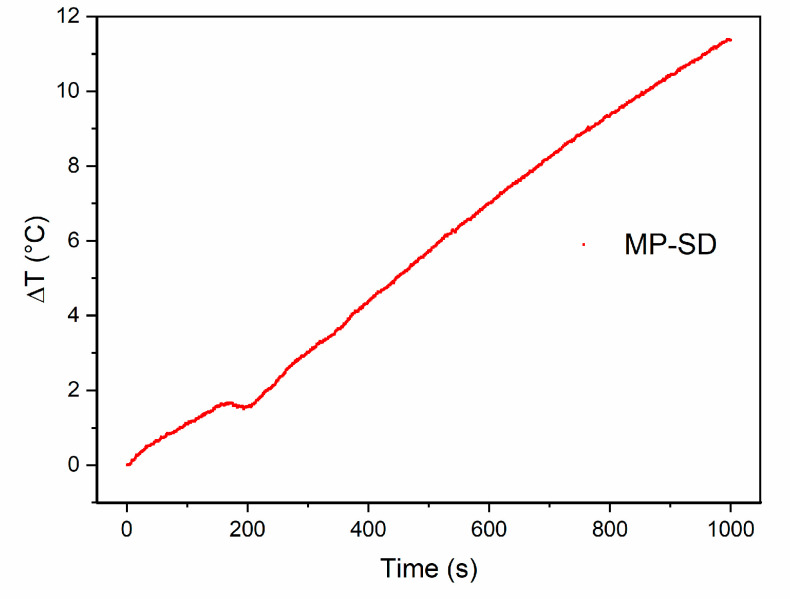
Hyperthermia results of temperature variation (ΔT) versus time of sample MP-SD, under magnetic field of 100 kHz and 25 mT.

A suspension of MP-SD particles was also submitted to an alternate magnetic field to check if it is able to produce heat suitable for magnetic hyperthermia treatment. At 10 mg/mL, under a field of 100 kHz and a 25 mT amplitude, the temperature was increased to almost 12 °C in 1000 s, as shown in Figure 7; usually, an increase of 5–8 °C is required at the site of the tumor, to reach 42–46 °C, which is sufficient to kill the cancer cells [8]. The concentration of particles, the field frequency and intensity, as well as the time of exposure to it, can be planned to get the desired temperature range at the site of application.

Cytotoxicity assays in vitro evaluated how the particles interfere in the growth and viability of healthy cells NCTC 929 and with tumor cells HeLa and HepG2, and therefore predict their potential toxic effects in whole animals, without the application of a magnetic field. In this study, high concentrations were tested in comparison to typical concentrations for cytotoxicity evaluation in the literature [37], in order to evaluate the range of concentrations typically used in hyperthermia studies, which can be higher than 1 mg/mL.

**Figure 8:**
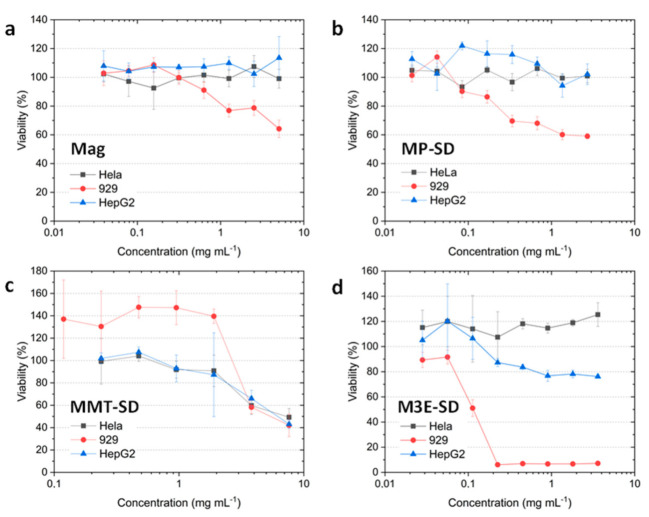
Viability of HeLa, HepG2, and 929 cell lines in contact with different concentrations of samples Mag (**a**), MP-SD (**b**), MMT-SD (**c**), and M3E-SD (**d**).

Bare magnetite (Figure 8a) did not affect the viability of both tumor cells, standing around 100% up to 5 mg/mL, while NCTC 929 is viable up to 0.3 mg/mL, diminishing to 79% with 2.5 mg/mL. MP-SD (Figure 8b) did not affect the viability of tumor cells up to 5 mg/mL as well, and NCTC 929 was 90% viable up to 0.1 mg/mL. M3E-SD (Figure 8d) was the sample that most affected NCTC 929, with viability of 90% up to 0.05 mg/mL and lower than 10% for more than 0.2 mg/mL.

MMT-SD (Figure 8c) results must be interpreted considering the influence of soluble maltodextrin and Tween since it was seen by DLS results that the particle erodes in water. Up to 1 mg/mL, the viability of NCTC 929 was around 140%, possibly due to an increase in nutrient availability due to maltodextrin release. For more than 1 mg/mL, the viabilities of all cell lines were between 40% and 80%, possibly due to a higher effect of Tween 80, that even though it is used in drug formulations, may be toxic in high concentrations [38].

## 4. Conclusions

Nano Spray Drying was presented as an interesting technique to encapsulate magnetite nanoparticles into polymeric matrixes, to form particles with average sizes around 1 µm, in a size range smaller than regular spray drying. It was shown how it can be used with several polymers and magnetite/polymer ratios, being a versatile strategy for different purposes. By just changing the type or amount of polymer used, one can get different characteristics, such as a fast erosion and drug release by using maltodextrin, a non-spherical particle shape by using Eudragit S100, or a higher magnetic saturation by using less polymer as in the MP-SD formulation with Pluronic. The obtained powders also have the superparamagnetic property typical of bare magnetite nanoparticles at room temperature, with lower blocking temperatures, which can be related to the presence of smaller magnetic nuclei (synthesized in the presence of polymers) and to lower magnetic coupling.

The sample encapsulated with Pluronic (MP-SD) had high magnetic saturation considering it is a composite (68.7 emu/g at 300 K and 79.5 emu/g at 1.7 K) and it was also submitted to an alternate magnetic field, which proved that it can produce heat for magnetic hyperthermia treatment of cancer. Furthermore, its cytotoxic evaluation showed that the three cell lines tested were at least 90% viable up to the concentration of 0.1 mg/mL, indicating the viability of its biomedical application.

## Figures and Tables

**Table 1:** Composition of the dispersions of magnetite and polymers used for nano spray drying.

Sample	Composition (Mass Ratios)	Total Concentration (*m*/*v*)
MP-SD	1 MP	0.8%
ME-SD	1 ME	0.8%
M3E-SD	1 ME: 2 Eudragit	0.8%
MTM-SD	1 MT: 2 Maltodextrin: 2 Tween 80	1.0%

**Table 2:** Particles’ average diameters determined by microscopy (SEM and TEM) and Z-average hydrodynamic diameter by dynamic light scattering (DLS), with polydispersity index (PdI) in parenthesis.

Sample	Particle Diameter (nm)		
	SEM	TEM	DLS (PdI)
Mag	-	10.5 ± 2.7	206.6 (0.457)
MP-SD	1016 ± 345	-	993.1 (0.178)
ME-SD	899 ± 310	-	812.3 (0.138)
M3E-SD	1066 ± 351	-	1392.3 (0.145)
MMT-SD	1155 ± 367	-	288.0 (0.262)

**Table 3:** Magnetic features of the samples: blocking temperature (T_B_), magnetic saturation (M_S_), intrinsic coercivity (Hc), ratio (%) between M_S_ of composite and pure magnetite (M_S-Composite_/M_S-Mag_), and TGA residues.

Sample	T_B_ (K)	M_S_ (emu/g)		Hc (Oe)		M_S-Composite_/M_S-Mag_	TGA Residue
		1.7 K	300 K	1.7 K	300 K		
Mag	210	83.1	72.0	282.0	7.5	-	95.8%
MP-SD	174	79.5	68.7	219.6	3.8	95.5%	93.6%
ME-SD	138	64.3	53.9	311.9	22.2	76.1%	85.3%
M3E-SD	149	42.1	35.8	301.2	2.2	50.2%	64.8%
MMT-SD	182	12.9	11.1	342.6	2.5	15.5%	20.7%

## Data Availability

All data presented in this article are presented here.
